# Honokiol Microemulsion Causes Stage-Dependent Toxicity Via Dual Roles in Oxidation-Reduction and Apoptosis through FoxO Signaling Pathway

**DOI:** 10.3390/cells11223562

**Published:** 2022-11-11

**Authors:** Hui Li, Wanfang Li, Jie Li, Sizheng Li, Lian Kuang, Fei Pang, Haiyan Jiang, Hongtao Jin, Xiaolan Bian

**Affiliations:** 1 Department of Pharmacy, Ruijin Hospital, Shanghai Jiaotong University School of Medicine, Shanghai 200025, China; 2 New Drug Safety Evaluation Center, Institute of Materia Medica, Chinese Academy of Medical Sciences, Beijing 100050, China; 3 Beijing Union-Genious Pharmaceutical Technology, Ltd., Beijing 100176, China; 4 NMPA Key Laboratory for Safety Research and Evaluation of Innovative Drug, Beijing 102206, China

**Keywords:** honokiol microemulsion, zebrafish, PC12 cell, toxicity, oxidation-reduction, apoptosis, *FoxO*

## Abstract

Honokiol, the main bioactive extract of *Magnolia officinalis,* exhibits extensive therapeutic actions. Its treatment for advanced non-small cell lung cancer is undergoing clinical trials in China. However, the published safety evaluation studies have focused on extract mixtures of *Magnolia officinalis* in which the honokiol content was well below the reported clinical dose of the honokiol monomer. Therefore, safety assessment of the honokiol monomer is urgently needed. Our previous studies have already demonstrated that a high dose of the honokiol microemulsion (0.6 μg/mL) induces developmental toxicity in rats and zebrafish by inducing oxidative stress. By exploring the relationship between time and toxicity, we found that developmental toxic responses were stage-dependent. They mainly occurred within the first 24 h post fertilization (hpf) especially the first 12 hpf. In zebrafish, low doses of honokiol microemulsion (0.15, 0.21 μg/mL) significantly decreased the levels of reactive oxygen species (ROS) and malondialdehyde (MDA) and increased the mRNA expression of *bcl-2.* In contrast, high dose (0.6 μg/mL) increased the levels of ROS and MDA, decreased activities and mRNA expression of superoxide dismutase (SOD) and catalase (CAT), and increased mRNA expression of *bax, c-jnk, p53* and *bim.* By acridine orange staining, we found that a high dose of honokiol microemulsion induced apoptosis mainly in zebrafish brain. In rat pheochromocytoma cells (PC12 cells), low doses of the honokiol microemulsion (1, 5, 10 µM) exerted a protective effect against H_2_O_2_-induced oxidative damage while high doses (≥20 µM) induced oxidative stress, which further confirms the dual effects of honokiol microemulsion on nerve cells. These dual roles of the honokiol microemulsion in oxidation–reduction reactions and apoptosis may be regulated by the forkhead box class O (FoxO) signaling pathway. Due to the potential of developmental toxicity, we recommend that the administration of high dose honokiol microemulsion in pregnant women should be considered with caution.

## 1. Introduction

**Figure 1:**
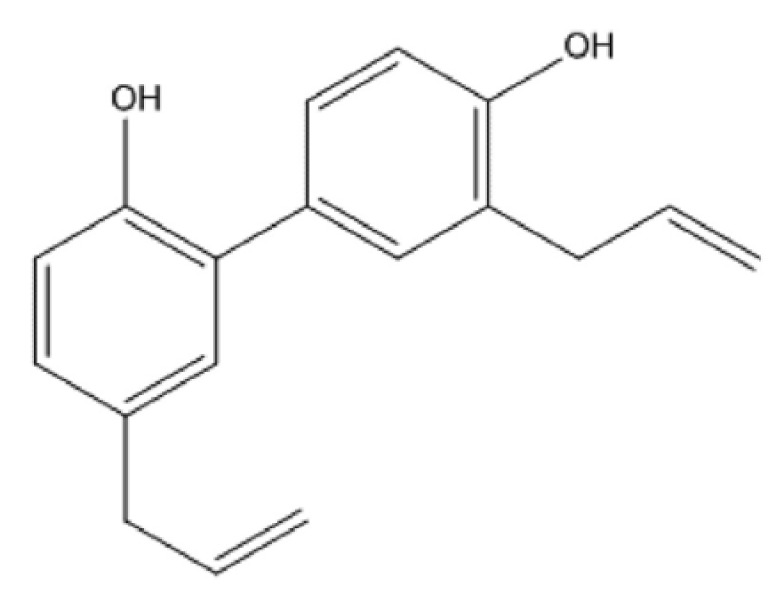
Chemical structure of honokiol.

*Magnolia plants,* as traditional herbal medicines, have been widely used in Asian countries such as China, Japan and Korea for the treatment of various diseases. Honokiol (C_18_H_18_O_2_, Figure 1), a phenylpropanoid molecule, is one of the main ingredients responsible for the beneficial properties of *magnolia* bark and leaf extracts. To date, numerous investigations have demonstrated that honokiol has wide-ranging pharmacological activities. The extensive therapeutic actions of honokiol include anticancer, antidepressant, antimicrobial, anxiolytic, antithrombotic, analgesic, antispasmodic, neuroprotective, cardioprotective, hepatoprotective, anti-inflammatory and antioxidative activities [1,2]. These activities are associated with multiple signaling pathways and molecular and cellular targets including nuclear factor-κB (NF-κB), epidermal growth factor receptor (EGFR), cell survival signaling, signal transducer and activator of transcription 3 (STAT3), the cell cycle, cyclooxygenase and other inflammatory mediators. Honokiol has broad chemopreventive and therapeutic effects on cancers of different organs [3] and is considered as a promising new drug for conventional drug-resistant cancers [4,5,6]. The highly lipophilic property of honokiol allows it to readily cross the blood-brain barrier and blood-cerebrospinal fluid barrier with high bioavailability. However, this property also limits its clinical usage due to low oral bioavailability and difficulty in intravenous administration. Fortunately, the development of various formulations of honokiol, including microemulsion, liposomes [7], nanoparticles [8] and micelle copolymers [9], have successfully solved the problem of low water solubility. Undoubtedly, honokiol has a bright future in clinical applications.

To guarantee a successful launch of this treatment, more attention should be given to safety evaluations of honokiol. By searching related publications, we found that safety evaluation studies have mainly concentrated on magnolia plant extracts including both honokiol and magnolol without examining the specific contents. The toxicity evaluation results of the mixed extracts of honokiol and magnolol are somewhat controversial both in animals [10,11,12] and human [13,14]. Regarding the honokiol monomer, only animal studies have been reported until now. In beagle dogs, the approximate lethal dose range was 66.7–100.0 mg/kg, and no adverse effects were observed below the dose of 0.25 mg/kg [15]. In Sprague-Dawley rats, the honokiol microemulsion has no toxic effects at a dose up to 500 µg/kg, according to our previous studies [16]. However, a high dose of honokiol microemulsion showed developmental toxicity in pregnant rats (2000 µg/kg/d) [16] and zebrafish embryos (0.6 µg/mL) [17]. By comparison, the reported antitumor dose for humans is much higher than these above reported maximum equivalent toxic dose for animals [18]. Consequently, further safety evaluations of high doses of honokiol are still needed to identify possible side effects.

The developmental toxicity of some chemicals, such as triclabendazole sulfoxide and albendazole, is stage-dependent [19,20]. Whether the embryonic developmental toxicity of honokiol is stage-specific or organ-specific has never been reported. Therefore, it is necessary to confirm whether the toxicity of the honokiol microemulsion in zebrafish embryos depends on developmental stages. The content of ROS is considered as a critical determinant of the toxicity of ionizing radiation and chemotherapeutic drugs. In addition to oxidative stress, several studies have demonstrated that excessive intracellular ROS generation also triggers apoptosis [21]. ROS-mediated apoptosis is a mechanism of some anticancer drugs [22,23]. Our previous study indicated that a high dose of honokiol microemulsion inhibited SOD enzyme activity and increased ROS levels, which subsequently caused embryo-developmental toxicity in zebrafish [17]. It is necessary to identify whether the embryonic developmental toxicity of the potential anticancer drug honokiol is associated with ROS-induced apoptosis. The reported mechanisms are that increases in ROS activate many confirmed transcription factors, including nuclear factor-erythroid 2 p45-related factor 2 (Nrf2), forkhead box class O (FoxO), activator protein 1 (AP-1), nuclear factor κB (NF-κB), heat shock factor 1 (HSF1) and tumor protein p53, to regulate the redox state of cells [24,25]. Among these transcription factors, FoxO mediates primary biological responses, including proliferation, apoptosis, differentiation, metabolism and oxidative stress, and is considered as an important sensor, mediator and regulator of redox signaling [26]. Therefore, we also explored the potential roles of FoxOs in the embryonic developmental toxicity of high-dose honokiol. In summary, we used zebrafish to further clarify the characteristics and mechanisms of the pharmacological and toxic actions of honokiol microemulsion. In addition, PC12 cells were employed to validate the antioxidation and pro-oxidation effects on the nervous system.

## 2. Materials and Methods

### 2.1. Chemicals

The honokiol microemulsion was a slightly yellow, oily liquid with a concentration of 10 mg/mL. High performance liquid chromatography showed that the honokiol microemulsion contained 9.7 mg/mL honokiol with low relative standard deviation and was stable [15,17]. In order to avoid light, it was stored in a brown bottle at a temperature of −20 °C. Professor Chen from the School of Pharmaceutical Sciences (Peking University, Beijing, China) generously donated the test material to us.

### 2.2. Zebrafish Husbandry

AB strain wild type adult zebrafish were maintained in a recirculating aquaculture system under a cycle of 14 h/10 h light/dark at 28 °C. The detailed fertilization, collection and selection processes of the zebrafish embryos were identical to those in our previously published article [17]. Normally developed embryos were selected and immersed in a medium containing honokiol microemulsion or in E3 medium (5 mM NaCl, 0.17 mM KCl, 0.33 mM CaCl_2_, and 0.33 mM MgSO_4_). All experimental embryos were maintained in a constant-temperature/constant-light incubator (Zhongyiguoke, Beijing, China). Every 24 h, the medium was changed and dead embryos or larvae were removed from the culture plates.

### 2.3. Honokiol Microemulsion Exposure

According to the developmental characteristics of zebrafish embryos and related publications, the following time points were critical in the development of the embryos: 3 hpf (1000-cell blastula period), 12 hpf (5-somite segmentation period), 24 hpf (prim-15 pharyngula period), and 96 hpf (larval stage; the endpoint of the acute toxicity test recommended by the Organization for Economic Co-operation and Development) [19,27,28]. Therefore, these selected time points were used to confirm the relationship between toxic effects and time. The immersion concentrations of the honokiol microemulsion (0.15, 0.21, 0.30, 0.60 and 0.84 μg/mL) were selected on the basis of our previously reported toxic range for zebrafish embryos [17]. The control embryos were exposed to E3 medium. During the experimental period (3–96 hpf), we evaluated the rate of mortality every 24 h and recorded the rate of hatching at 72 and 96 hpf. At 96 hpf, notochord malformation, pericardial edema and yolk sac edema were considered as malformations of larvae, and were assessed using a three-dimensional microscope (Keyence, Osaka, Japan). Except for those mentioned above that were used to explore the time-response relationship, zebrafish embryos used in other experiments were exposed to the honokiol microemulsion or E3 medium from 3 to 96 hpf.

### 2.4. Determination of Oxidation Product Content and Antioxidant Enzyme Activity Levels

We analyzed the content of oxidation products and the activity of antioxidant enzymes using embryos/larvae at 24 and 96 hpf. The generation of ROS and MDA were measured by the chemiluminescence method and thiobarbituric acid (TAB) method, respectively, using assay kits (Nanjing Jiancheng Bioengineering Institute, Nanjing, China). The activity of CAT was assayed by the ammonium molybdate method using assay kits from Nanjing Jiancheng Bioengineering Institute. The activity of SOD was measured by an indirect method based on xanthine oxidase and a novel color reagent using an assay kit from Sigma. These assays were performed in strict accordance with the manufacturer’s instructions. All test tissues were prepared according to descriptions in our previous publications [17]. All tests were repeated three times.

### 2.5. Analysis of Gene Expression

Approximately 40 homogenized zebrafish embryos/larvae at 24/96 hpf that had been exposed to the honokiol microemulsion or E3 medium were used to analyze mRNA expression. Total RNA of fresh measured samples was firstly extracted using an SV total RNA isolation system kit (Promega, Madison, WI, USA), and then cDNA was synthesized using a GoScript^TM^ Reverse Transcription system kit (Promega). Lastly, we performed the quantitative determinations of the amplification products by fluorescence quantitative polymerase chain reaction (PCR) using the GoTaq^®^ qPCR master mix kit (Promega) in an Applied Biosystems 7500 Real-Time PCR System. All kits were used in accordance with the manufacturer’s instructions. Zebrafish-specific primers for the genes of interest were designed using Primer 6.0 software or were obtained from previously reported data (Supplementary Material Table S1). The β-actin gene as a housekeeping gene was used as an internal control to standardize the results by eliminating variations in mRNA and cDNA quantity and quality [17,29]. The level of mRNA was calculated as the ratio to the β-actin mRNA level. For every selected gene, all reactions were performed on three replicate samples.

### 2.6. In Vivo Rapid Apoptosis Assay

As reported, the vital dye acridine orange is commonly used to quantify apoptosis in live zebrafish embryos [30,31]. Acridine orange is a nucleic acid selective metachromatic dye. It can diffuse through the cell membrane and bind with DNA and RNA. In apoptotic cells, yellow-green fragments were clearly observed under the fluorescence microscope. From 3 hpf, this rapid apoptosis assay was conducted at 24, 48, 72 and 96 hpf in living embryos exposed to the honokiol microemulsion. The vital dye was dissolved in E3 medium. Test samples were immersed in acridine orange at a concentration of 5 µg/mL for 10 min at room temperature. After staining, we repeatedly washed the living embryos 3 times with E3 medium. Then they were anesthetized with tricaine for quick imaging with a fluorescence microscope to avoid bleaching.

### 2.7. PC12 Cell Culture and Treatment

We purchased PC12 cells from the cell bank of the Chinese Academy of Science. The cells were cultured in complete DMEM containing 10% heat-inactivated horse serum and 5% fetal bovine serum in a humidified incubator of 5% CO_2_ in air at 37 °C. The PC12 cells were seeded in 96-well plates for 48 h and then were transferred into medium containing 50 ng/mL nerve growth factor (NGF) for 72 h. In the presence of NGF, PC12 cells can differentiate to morphologically resemble neurons with neuron-like functions, such as neurotransmitter release [32]. During the period of NGF stimulation, we observed and evaluated the differentiation of PC12 cells every 24 h.

For H_2_O_2_ treatment, the differentiated PC12 cells were cultured in ten concentrations including 0, 100, 200, 300, 400, 500, 600, 700, 800, and 900 μM for 12 h to determine the appropriate concentration for the cytotoxicity model.

For honokiol microemulsion treatment, differentiated PC12 cells were cultured in a series of concentrations (0, 1, 2.5, 5, 10, 20, 40, 60, 80, and 100 μM) for 12 h to determine the cytotoxic range.

### 2.8. MTT Assay

Cell viability was evaluated by the method of 3-(4,5-dimethylthiazol-2-yl)-2,5-diphenyltetrazolium bromide (MTT) colorimetric assay. PC12 cells in the logarithmic growth phase were placed in 96-well plates at concentrations of 5 × 10^3^, 1 × 10^4^, 2.5 × 10^4^, 5 × 10^4^ and 1 × 10^5^ cell/mL. In order to draw a cell growth curve, the MTT assay was performed from the first day to the seventh day. According to the cell growth curve, the appropriate seeding concentration was determined.

### 2.9. Statistical Analysis

All statistical analyses were performed using SPSS software (Version 25; SPSS, Inc., Chicago, IL, USA). All data are expressed as the mean ± standard deviation (SD) and were assessed for significance using one-way analysis of variance (ANOVA) with post hoc Tukey’s test. For all statistical tests, differences were considered significant at *p* < 0.05.

## 3. Results

### 3.1. The Developmental Toxicity of the Honokiol Microemulsion on Zebrafish Embryos Was Stage-Dependent

**Figure 2:**
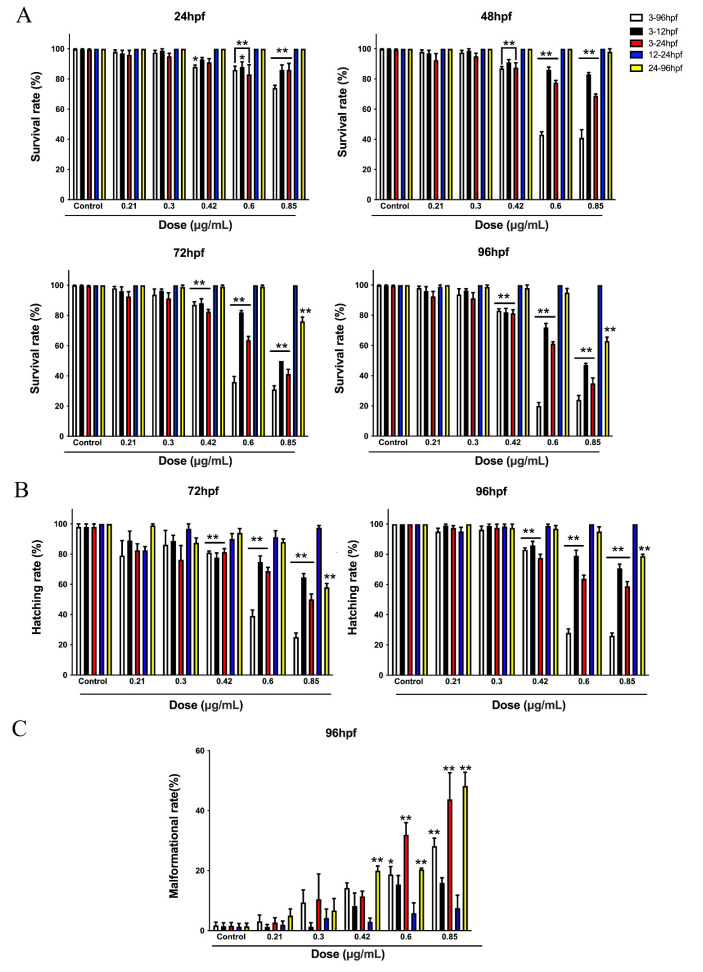
Relationship between the honokiol microemulsion exposure period and the toxic response. (**A**) Survival rates of zebrafish embryos at 24, 48, 72 and 96 hpf. (**B**) Hatching rates of zebrafish embryos at 72 and 96 hpf. (**C**) Malformation rates of zebrafish at 96 hpf. The data are expressed as the mean ± SD (* *p* < 0.05, ** *p* < 0.01 compared with control embryos).

With the aim of exploring the toxic response-‘time relationship, we divided zebrafish embryos into 5 groups, including the 3–96 hpf, 3–12 hpf, 3–24 hpf, 12–24 hpf and 24–96 hpf groups, according to the honokiol microemulsion exposure period. The exposure concentrations were 0 (control), 0.21, 0.3, 0.42, 0.6 and 0.85 µg/mL. In Figure 2, except for 24 and 48 hpf survival rates of some embryos exposed to 0.42 μg/mL, the embryos exposed to honokiol microemulsions of 0.42, 0.6 and 0.85 μg/mL for 3–96 hpf, 3–12 hpf, and 3–24 hpf showed statistically decreased survival rates at 24, 48, 72, and 96 hpf and reduced hatching rates at 72 and 96 hpf compared with the control groups, respectively. The rates of malformation of the embryos exposed to 0.6 and 0.85 µg/mL for 3–96 hpf, 3–24 hpf and to 0.42 µg/mL for 24–96 hpf were significantly higher than those of the control embryos. Embryos exposed to 0.85 µg/mL for 24–96 hpf showed decreased survival rates and hatching rates at 72 and 96 hpf and increased malformation rates. However, the embryos exposed to all concentrations of the honokiol microemulsion for 12–24 hpf and 24–96 hpf exhibited no toxic effects on survival, hatching or morphology except the maximum concentration of 0.85 µg/mL for 24–96 hpf. Taken together, these findings indicate that the developmental toxic response mainly occurred within the first 24 hpf, especially within the first 12 hpf, and was also associated with the exposure concentration.

### 3.2. Dual Roles of the Honokiol Microemulsion in Oxidative Stress in Zebrafish Embryos

**Figure 3:**
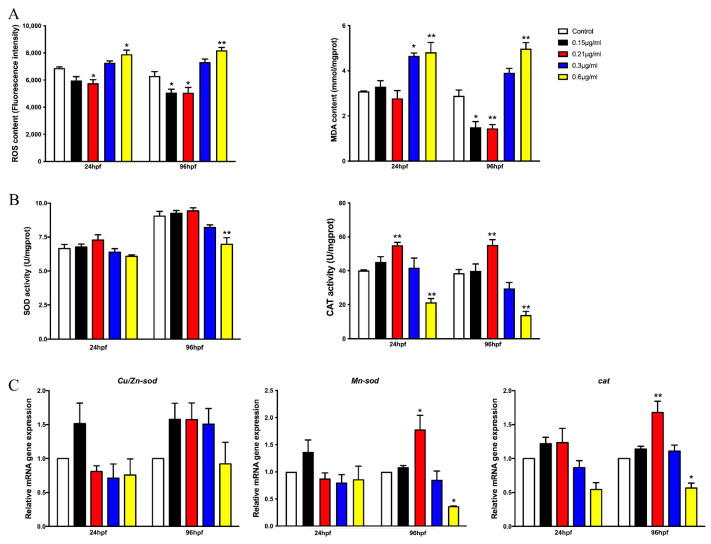
Measurement of the effect of the honokiol microemulsion on oxidative stress in zebrafish embryos. The content of oxidation products is shown in (**A**). The antioxidant activity is shown in (**B**). The mRNA expression of oxidative genes is shown in (**C**). The data are expressed as the mean ± SD (* *p* < 0.05, ** *p* < 0.01 compared with the control group).

Our previous publication demonstrated that the honokiol microemulsion exerts dual effects of antioxidation and triggering oxidative stress during the development of zebrafish embryos at 48 and 96 hpf [17]. The results regarding the toxic response-time relationship showed that the exposure period of the first 24 hpf, especially the first 12 hpf, was closely related to developmental toxicity. Consequently, we complementarily measured the oxidative stress response of honokiol microemulsion-exposed embryos at 24 hpf. The results showed that the levels of ROS and MDA were significantly decreased at a concentration of 0.21 μg/mL and increased at a concentration of 0.6 μg/mL in both 24 and 96 hpf embryos (Figure 3A). Embryos exposed to the honokiol microemulsion at 0.6 µg/mL showed reduced activity of SOD and mRNA expression of *Mn-sod* at 96 hpf (Figure 3B,C). The activity of CAT was increased at 0.21 µg/mL but decreased at 0.6 µg/mL in both 24 and 96 hpf embryos. The expression of *cat* mRNA was in accordance with the activity of CAT but was statistically significant only in 96 hpf embryos (Figure 3B,C).

### 3.3. The Honokiol Microemulsion Induced Apoptosis Mainly in the Zebrafish Brain

**Figure 4:**
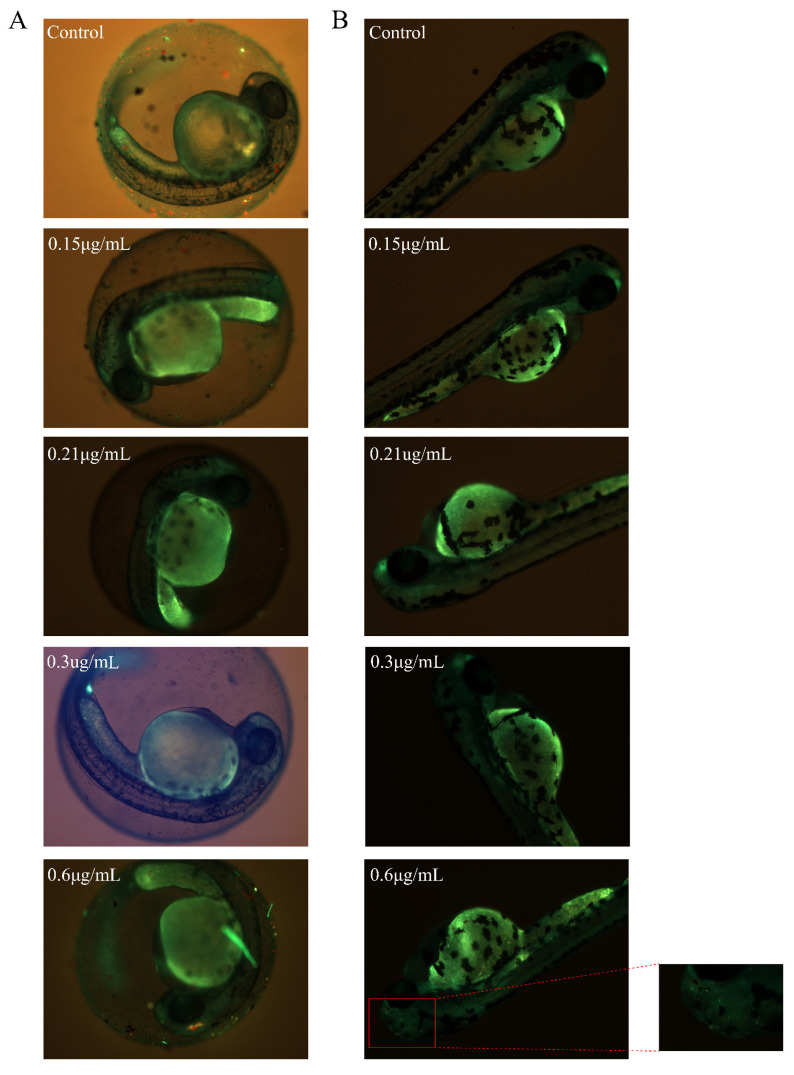

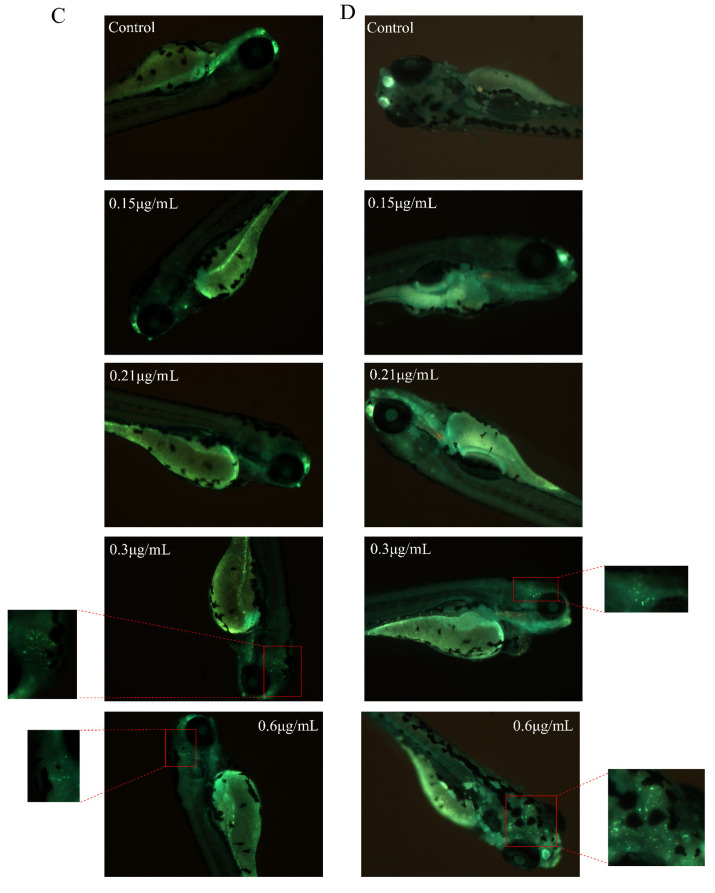
Representative images of whole-embryo cell death determined by acridine orange staining at 24 hpf (**A**), 48 hpf (**B**), 72 hpf (**C**), and 96 hpf (**D**) in the control and honokiol microemulsion-exposed groups. The red boxes indicate representative apoptotic cells in brain.

As reported, increases in ROS levels can subsequently induce apoptosis. A high dose of the honokiol microemulsion resulted in increased ROS levels. Therefore, we explored apoptosis in living zebrafish embryos using acridine orange staining. At 24 hpf, no apoptotic cells were detected in zebrafish embryos at any exposure concentration (Figure 4A). At 48, 72, and 96 hpf, embryos exposed to the maximum concentration of 0.6 µg/mL exhibited obvious apoptosis mainly in the brain (Figure 4B). With prolonged exposure time, embryos exposed to the honokiol microemulsion at 0.3 μg/mL also showed apparent apoptosis in the brain at 72 and 96 hpf (Figure 4C,D). These results correspond to the reduced locomotion in zebrafish exposed to a high concentration of the honokiol microemulsion [17].

### 3.4. The Honokiol Microemulsion Influenced the mRNA Expression of Apoptosis-Related Genes and Regulatory Genes in Zebrafish Embryos

**Figure 5:**
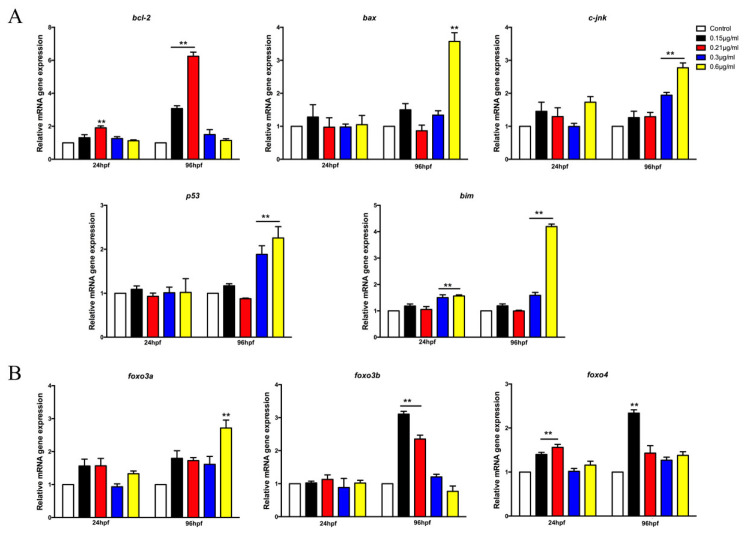
mRNA expression of apoptosis-related genes and regulatory genes in 24 and 96 hpf embryos after honokiol microemulsion treatment. The mRNA expression levels of antiapoptotic and proapoptotic genes are shown in (**A**). The mRNA expression levels of apoptosis regulatory FoxO subtypes are shown in (**B**). The data are expressed as the mean ± SD (** *p* < 0.01 compared with control).

With the aim of further confirming the relationships between apoptosis and the honokiol microemulsion, we measured the mRNA expression of apoptosis-related genes and regulatory genes in zebrafish embryos. The gene expression of apoptosis suppressor *bcl-2* was significantly increased in 24 and 96 hpf embryos that were exposed to the honokiol microemulsion at a concentration of 0.21 µg/mL. The expression of *bax* was only upregulated in the 0.6 µg/mL group at 96 hpf. In the 0.3 and 0.6 µg/mL groups, the mRNA expression levels of the proapoptotic genes *c-jnk* and *p53* were significantly decreased at 96 hpf, and the expression of *bim* was significantly upregulated at both 24 and 96 hpf (Figure 5A). Regarding apoptosis regulatory genes, the mRNA expression levels of *foxo3a* were increased at 0.6 µg/mL and those of *foxo3b* and *foxo4* were increased at exposure concentrations of 0.15 and 0.21 µg/mL.

### 3.5. Dual Roles of the Honokiol Microemulsion in Oxidation-Reduction in PC12 Cells

**Figure 6:**
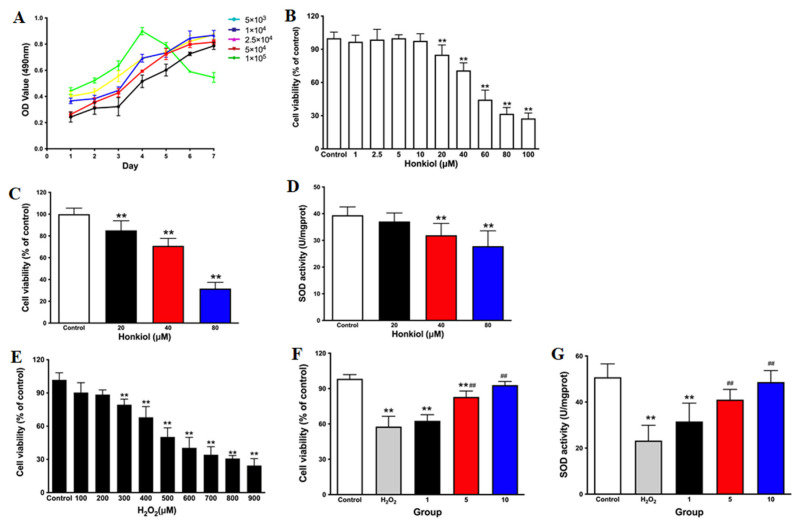
Dual roles of the honokiol microemulsion in oxidation-reduction in PC12 cells. (**A**) Cell growth curves of PC12 cells at different inoculation concentrations. (**B**) Effects of honokiol microemulsion on the viability of PC12 cells. (**C**) Effect of different toxic doses of honokiol microemulsion on the activity of PC12 cells. (**D**) Activity of SOD in PC12 cells exposed to different toxic doses of the honokiol microemulsion. (**E**) Effects of H_2_O_2_ on the viability of PC12 cells. Protective effects of the honokiol microemulsion on the cell viability (**F**) and SOD activity of PC12 cells injured by H_2_O_2_ (**G**). The data are expressed as the mean ± SD (** *p* < 0.01 compared with the control group; ^##^ *p* < 0.01 compared with the H_2_O_2_ model group).

After treatment with NGF, the differentiation and development of PC12 cells is similar to that of neurons; thus, cells treated in this way are considered as a verified model system for neuroscience research. We first confirmed the appropriate PC12 cell seeding density for 96-well plates with an MTT assay. The cell growth curve (Figure 6A) showed that the appropriate inoculation concentrations were 5 × 10^3^–5 × 10^4^ cells/mL. We selected 2.5 × 10^4^ cells/mL as the inoculation concentration for our following study. After exposure to the honokiol microemulsion, the observed minimal toxic dose for PC12 cells was 20 µM. When the exposure concentration was increased to 60 µM, the cell activity decreased by 50 percent (Figure 6C). The activity of SOD showed only a slight reduction at 20 µM but was significantly reduced at 40 and 80 μM (Figure 6D). These results further demonstrated that the toxic effect of the honokiol microemulsion was associated with oxidative stress. To further validate the antioxidation effect of treatment with a low dose of the honokiol microemulsion, we established an oxidative damage model in PC12 cells. The cells were exposed to a series of H_2_O_2_ concentrations ranging from 0 to 900 µM. We considered the suitable exposure concentration of H_2_O_2_ to be 500 µM, which led to a 50% reduction in cell viability (Figure 6E). Cells were successively exposed to 500 µM H_2_O_2_ for 12 h and 0–10 µM honokiol microemulsion for 12 h. Compared with those of the control group, the cell viability and SOD activity of the H_2_O_2_-damaged model and 1 µM honokiol microemulsion groups were significantly decreased (Figure 6F). Compared with those of the H_2_O_2_-damaged model group, the cell viability and SOD activity of the 5 and 10 µM honokiol microemulsion groups were significantly increased (Figure 6G).

## 4. Discussion

The extensive pharmacological activities of honokiol in animals, notably its regulation of oxidative stress, give honokiol to have the potential to be used as a therapeutic drug in a series of diseases. These may include myocardial ischemia/reperfusion injury in type 1 diabetes, dermatologic disorders, hepatic steatosis, Parkinson’s disease, sepsis-associated acute kidney/lung injury, and surgery-/anesthesia-induced cognitive decline [33,34,35,36,37,38,39,40]. In accordance with prior publications, we also demonstrated the antioxidant action of a low dose of the honokiol microemulsion in both zebrafish embryos and PC12 cells. However, human studies on honokiol have rarely been reported. The clinical trials databases of the NIH, WHO and China contain only one phase I clinical trial of honokiol that is ongoing for advanced non-small-cell lung cancer in China (CTR20170822). Most published studies have concentrated on complicated mixtures containing honokiol. One pilot double-blind placebo-controlled clinical trial evaluating the effect of a proprietary magnolia and philodendron extract (Relora) showed that Relora may somewhat relieve mild transitory anxiety in overweight premenopausal females without significant adverse events [13]. Another six-month clinical study on dentifrice containing 0.3% magnolia extract indicated that it significantly reduced gingivitis more than the corresponding control dentifrice [41]. It is important to note that these published clinical trials have concentrated on magnolia extracts including both honokiol and magnolol. Two clinical studies of 16 and 42 healthy premenopausal women, respectively, have reported adverse reactions, including heartburn, shaking hands, perilabial numbness, sexual dysfunction, thyroid dysfunction, fatigue and headaches in two participants at the maximum honokiol dosage of 11.25 mg/d [13,14]. A case report of two cancer patients who were intravenously administered honokiol at an initial dose of 10 mg/kg body weight that was subsequently gradually increased to 50 mg/kg based on individual tolerance over two weeks reported improvements in symptoms and quality of life accompanying transient sedation and minor nausea without adverse effects on blood count, liver function and kidney function [18]. By comparing doses, it is not difficult to find that the actual administration doses of honokiol in single preparations are much higher than those in compound extracts. This may explain why the toxic results observed with the compound extract that contains honokiol and the honokiol single preparations are somewhat controversial. The toxic doses of honokiol microemulsion in the present study and our previous studies were respectively 2000 µg/kg/d in pregnant rats [42], 0.6 µg/mL in zebrafish embryos [17] and 20 µM in PC12 cells. It may be mentioned that in our previous study of pregnant rats, sodium salicylate (250 mg/kg/day) was selected as the positive drug; this better proved the developmental toxicity of the honokiol microemulsion. Therefore, we only set a negative control of the culture medium of zebrafish embryos (E3 medium). According to the human rat equivalent dosage conversion, the potential toxic dose in humans may be 320 µg/kg/d. Thus, the actual administration doses of honokiol are much higher than the equivalent toxic dose. More attention should be paid to the safety evaluation of high dose honokiol. Considering the species differences between animals and humans, more clinical trials are still required to further verify our results.

Zebrafish, as a well-recognized biological model system, has been considered as an useful and efficient tool not only for toxicology but also for drug discovery [43,44]. More and more researchers extensively use zebrafish to evaluate the accumulation and toxicology of microplastics and nanoplastics which are an emerging threat to humans and the environment [45,46]. Compared with conventional mammals, zebrafish offers many compelling advantages including transparency of the embryo, short test period, lower costs and higher throughput. Moreover, the processes of embryogenesis in zebrafish are very similar to those in higher vertebrates, including humans. Published toxicity results for zebrafish embryos show consistency with those for mammals. Interestingly, various chemicals, such as ketamine, ethanol, albendazole and lead, are reported to cause stage-dependent developmental toxicity in zebrafish embryos [19,27,47,48]. Notably, there are variations in the periods of sensitivity to different toxicants. With the aim of confirming the period of sensitivity to the honokiol microemulsion, we employed zebrafish embryos to continually investigate the relationship between time and developmental toxicity. The results indicate that the sensitive time window of developmental toxicity of the honokiol microemulsion in zebrafish embryos is the first 24 hpf, especially the first 12 hpf. In the first 24 hpf, all major organs of zebrafish are formed. According to research on the effects of ethanol exposure on zebrafish embryogenesis, the 6–24 hpf, 24–48 hpf and 48–72 hpf periods of zebrafish are approximately equivalent to the first, second, and third trimesters of pregnancy in humans, respectively [49]. We assume that the first 24 hpf of zebrafish corresponds to the first 3 months of pregnancy. Consequently, we suggest that the use of the honokiol microemulsion in pregnancy, especially during the initial and critical period of human embryo development and organogenesis, should be cautiously implemented or forbidden.

In zebrafish, nervous system development starts at approximately 6 hpf. At the end of gastrulation (9–10 hpf), morphogenetic convergence movements have shaped the neural plate into a tube. In the following 6 h, the neural tube is subdivided into separate brain regions. By 24 hpf, the brain is already divided into the forebrain, midbrain, hindbrain and spinal cord, and the earliest clusters of neurons are interconnected. By 48 hpf, the brain ventricles have been formed, and the embryo can respond to some stimuli. At 96 hpf, the larvae have the same swimming rate as adult zebrafish and display advanced neuron functions with the formation of various glial cells [50]. The honokiol exposure period in our study on apoptosis was from 3 to 96 hpf, which covered the entire development of the nervous system. As mentioned above, the most sensitive time windows for different toxicants are variable. Djai and Rhiannon have found that environmental toxicant exposure is closely associated with neurodevelopmental disorders and that the majority of time windows cover prenatal periods [51]. We did not elaborately study the sensitive time windows of neurodevelopment toxicity of the honokiol microemulsion, which was a limitation of this study. But we observed apoptosis in the brain of zebrafish at 72 and 96hpf. In agreement with our results, neuroblastoma cells exposed to honokiol for 24 h can induce autophagic apoptosis by activating a p53-dependent mechanism [52]. The mRNA expression of proapoptotic genes, including *bax*, *c-jnk*, *p53* and *bim*, were upregulated at a high dose of the honokiol microemulsion (0.6 µg/mL) and the antiapoptotic gene *bcl-2* was upregulated at low doses (0.15, 0.21 µg/mL). The dual roles in apoptosis at different doses may be associated with pharmacological action and toxicity of honokiol.

In addition to apoptosis, this and our previous studies have found that high doses of the honokiol microemulsion increase the content of ROS at 24, 48 and 96 hpf and low doses enhance the activity of antioxidases. Excessive intracellular ROS generation subsequently activates many confirmed transcription factors to induce oxidative stress and apoptosis. Therefore, the observed apoptosis in the nervous system may have been triggered by increased ROS levels. Some experts consider that FoxO proteins are essential for oxidative stress to result in apoptosis via modification of protein interactions. In order to coordinate responses to environmental changes, FoxOs are transported from the cytoplasm to the nucleus and acetylated to mediate many primary biological responses, including apoptosis, proliferation, differentiation, and metabolism [53,54]. In Alzheimer’s disease, oxidative stress also promotes the transcriptional activity of FoxO, leading to increased production of ROS. This vicious cycle further brings about neural apoptosis and loss [55]. In humans and mammals, there are four *foxo* genes: *foxo1*, *foxo3a*, *foxo4* and *foxo6*. *foxo2* is homologous to *foxo3*, and *foxo5*, also known as *foxo3b*, is expressed only in zebrafish [56]. FoxO proteins are highly expressed in neural stem cells but selectively expressed in the nervous system with diverse biological functions. Therefore, FoxO proteins are increasingly being identified as potential targets for diseases of the nervous system [57,58]. During the embryonic development period of zebrafish, knockdown of *foxo3a* increases neuronal apoptosis, resulting in neural development defects, which indicates that *foxo3a* might be essential for the maintenance of neural development [59]. By RT-PCR, we found that the mRNA expression of *foxo3a* was upregulated at a high dose (0.6 µg/mL) and that the expression of *foxo3b* and *foxo4* was upregulated at low doses of the honokiol microemulsion (0.15, 0.21 µg/mL) in zebrafish embryos. In addition, Halasi and his colleagues also found that honokiol is a FoxM1 antagonist that inhibits FoxM1-mediated transcription and FoxM1 protein expression [60].Consequently, our subsequent research will further explore the exact mechanism and signaling pathway of FoxOs and the roles of FoxM1 in the pharmacological and toxic effects of honokiol.

## 5. Conclusions

In this study, we used zebrafish and PC12 cells in combination to further validate the dual roles of the honokiol microemulsion in redox reactions and apoptosis. In zebrafish embryos, low doses of the honokiol microemulsion (0.15, 0.21 µg/mL) exerted antioxidative effects by increasing the activity of antioxidant enzymes and antiapoptotic effects by upregulating the mRNA expression of *bcl-2* in zebrafish embryos. A high dose of the honokiol microemulsion (0.6 µg/mL) induced developmental toxicity by increasing the content of ROS and MDA; decreasing the activity of SOD and CAT; and upregulating the mRNA expression of proapoptotic genes, including *bax, c-jnk, p53* and *bim*. The developmental toxicity exhibited two characteristics: stage-dependency, with a sensitive time window of the first 24 hpf, especially the first 12 hpf, and nervous system specificity of apoptosis. The transcription factors FoxO3a, FoxO3b and FoxO4 may play regulatory roles in the pharmacological and toxic effects of the honokiol microemulsion. In PC12 cells, a low dose of the honokiol microemulsion (1–10 µM) exerted a protective effect against H_2_O_2_-induced oxidative damage by upregulating the activity of SOD. A high dose of the honokiol microemulsion (≥20 µM) induced cytotoxicity by decreasing the activity of SOD. Consequently, we suggest that clinical administration of high doses of honokiol microemulsion in pregnancy, especially during the initial and critical period of human embryo development and organogenesis, should be cautious.

## Data Availability

All data used to support the finding of this study are contained within the article.

## Supplementary Materials

The following supporting information can be downloaded at: https://www.mdpi.com/article/10.3390/cells11223562/s1, Table S1: Primers used in the PCR reactions.
