# Supplementary material for: Honokiol Microemulsion Causes Stage-Dependent Toxicity Via Dual Roles in Oxidation-Reduction and Apoptosis through FoxO Signaling Pathway
Source: Cells. 2022 Nov 11;11(22):3562. doi: 10.3390/cells11223562 (PMC9688712; doi:10.3390/cells11223562)
Supplement: Supplementary file 1 [file cells-11-03562-s001.zip › cells-1993481-supplementary.pdf]

Supplementary Data

Table S1. Primers used in the PCR reactions.

Gene	Sequences(5’-3’)
β-actin	F: CAAAGGGAGGTAGTTGTCTAACAGG<br>R: TTGTGAGGAGGGCAAAGTGG
Mn-sod	F: TTCAGGGCTCAGGCTGG<br>R: ATGGCTTTAACATAGTCCGGT
Cu-zn sod	F: GGCCAACCGATAGTGTTAGA<br>R: CCAGCGTTGCCAGTTTTTAG
Bcl-2	F: TCAATAAAGCAGTGGAGGAATC<br>R: TCAAATGAGGGTCTGAACGAG
bax	F: GGCTATTTCAACCAGGGTTCC<br>R: TGCGAATCACCAATGCTGT
c-jnk	F: TGGATACAACCACAAGGCTCT<br>R: GTCACGTTCTTGGGACACAG
P53	F: GGGCAATCAGCGAGCAAA<br>R: ACTGACCTTCCTGAGTCTCCA
bim	F: TGTGCCAGGTTCCCCTCTAA<br>R:AGTGCTGGTGGCTATGTTCTGAT
Foxo3a	F: AACATCAGCTTGACGGCTTC<br>R: GGAGGGCTAAACAGGGAGTT
Foxo3b	F: CCAAGCACCTCTACATCTC<br>R: CTGTGAGAGACCAGCGAAT
Foxo4	F: ATGGAGGAAGAAAACGTACCCC<br>R: TTAGCTGGGCACCCAGTTG
